# Synthesis and Characterization of Calcium Carbonate Obtained from Green Mussel and Crab Shells as a Biomaterials Candidate

**DOI:** 10.3390/ma15165712

**Published:** 2022-08-19

**Authors:** Rifky Ismail, Tezara Cionita, Wong Ling Shing, Deni Fajar Fitriyana, Januar Parlaungan Siregar, Athanasius Priharyoto Bayuseno, Fariz Wisda Nugraha, Rilo Chandra Muhamadin, Ramli Junid, Nor Azam Endot

**Affiliations:** 1 Department of Mechanical Engineering, Faculty of Engineering, Diponegoro University, Semarang 50275, Indonesia; 2 Faculty of Engineering and Quantity Surveying, INTI International University, Nilai 71800, Malaysia; 3 Faculty of Health and Life Sciences, INTI International University, Nilai 71800, Malaysia; 4 Department of Mechanical Engineering, Universitas Negeri Semarang, Kampus Sekaran, Gunungpati, Semarang 50229, Indonesia; 5 College of Engineering, Universiti Malaysia Pahang, Gambang 26300, Malaysia; 6 Department of Chemistry, Faculty of Science, Universiti Putra Malaysia, Serdang 43400, Malaysia

**Keywords:** mussel and crab shells, precipitation, calcium carbonate, bioceramic, biomedical

## Abstract

Green mussel and crab shells are natural sources of CaCO_3_, which is widely used as a bioceramic for biomedical applications, although they are commonly disposed of in landfills. The improper disposal of green mussel and crab shells can cause environmental pollution, reducing the quality of life in the community. Many studies have reported the preparation of CaCO_3_ from green mussels and crab shells. However, there are limited studies comparing the characteristics, including the crystal phase obtained, weight percentage (%) of crystal, crystal size, crystal system, and elemental composition of CaCO_3_ from green mussel shells, crab shells, and commercial CaCO3. The objective of this research was to compare the calcium carbonate properties formed from green mussel (PMS) and crab (PCS) shells to commercial CaCO_3_. Green mussel and crab shells were crushed to powder and were calcined at 900 °C for 5 h. Precipitated Calcium Carbonate (PCC) was synthesized from calcined green mussel and crab shells using a solution of 2M HNO_3_, NH_4_OH, and CO_2_ gas. The effect of setting parameters on the synthesized product was analyzed using XRD and SEM-EDX methods. This study shows that the chemical composition of PMS is nearly identical to that of commercial CaCO_3_, where no contaminants were identified. In contrast, PCS has N components other than Ca, C, and O. Furthermore, the predominance of the vaterite crystal phases in PMS and PCS, with respective weight percentages of 91.2% and 98.9%, provides a benefit for biomaterial applications. The crystallite sizes of vaterite in PMS, PCS, and calcite in commercial CaCO_3_ are 34 nm, 21 nm, and 15 nm, respectively.

## 1. Introduction

Shellfish are animals that live in water (salt water or fresh water) and have a shell or shell-resembling exterior. Commonly, shellfish can be divided into crustaceans and mollusks [1]. Crustaceans are aquatic animals that have jointed legs, a hard shell, and no backbone, such as crabs, crayfish, lobsters, prawns, and shrimp. Most mollusks have a hinged two-part shell, including clams, green mussels, oysters, and scallops, and various octopuses, snails, and squids. Shellfish has been a popular and favorite food in ancient and modern civilizations.

This has occurred because fresh shellfish is an excellent source of protein and a good source of minerals for humans. Additionally, most shellfishes are low in fat, cholesterol, and sodium [1].

Green mussels and crabs are examples of shellfishes widely consumed and traded by Indonesians, such as in Central Java. In 2019, the production of green mussels and crab in Central Java reached 12,500 tons [2] and 161 tons [3], respectively, to meet domestic and export needs. The green mussel shells and crab shells represent 70% [4] and 50% [5] of the total production, so the Province of Central Java produced 8750 tons of green mussel shells and 80.5 tons of crab shells in 2019. The results of these calculations show that the waste of green mussels and crab is very abundant and easy to find in the province of Central Java.

The improper disposal of solid waste in the form of green mussel shells and crab shells can cause environmental pollution, reducing the quality of life of the community. Green mussel and crab shells, including seashells, are very rich in calcium carbonate [4]. Nanoparticles such as calcium carbonate found in seashells are the main target of researchers’ applications in the biomedical field [6]. Currently, the main production of calcium carbonate comes from geological sources, with market prices varying from USD 60 per ton for coarse particles to USD 250 per ton for fine particles. However, calcium carbonate from geological sources has the risk of containing heavy metals, which are harmful to humans. Meanwhile, calcium carbonate from seashells such as green mussel and crab shells is relatively safe for human consumption and medical applications. The market price of medical-grade calcium carbonate can be as high as USD 1600 per ton [7].

PCC stands for Precipitated Calcium Carbonate, also known as purified, refined, or synthetic calcium carbonate. In general, PCC is when calcium carbonate is hydrated and then reacted with carbon dioxide. PCC has a high economic value because it has advantages such as size distribution, crystal phase, and specific surface area, which can be controlled during the synthesis process. Due to its excellence, PCC is widely applied in the pharmaceutical, biological, and biomedical fields [8]. Calcium carbonate from seashells such as green mussel and crab shells as a raw material for bioceramics for biomedical applications provides economic and environmental benefits. Economic advantages are due to the low price of waste, the availability in large quantities, and the ease of obtainment. Meanwhile, the use of waste materials directly can reduce environmental problems [9]. Bioceramics of seashells in biomedical applications are used for bone graft substitutes [10,11,12], dental materials [5,13,14], and drug delivery systems [6,15,16,17]. Calcium phosphate (CaP) bioceramics such as hydroxyapatite (HA) and tricalcium phosphate (TCP) are used for bone substitutes because they have excellent biocompatibility and osteoconductive properties [18]. Therefore, the objective of the present study is to compare the physical properties of commercial calcium carbonate and calcium carbonate synthesized from green mussel and crab shells that are easily found in the Central Java Province, Indonesia.

## 2. Materials and Methods

The green mussel and crab shells in this study were obtained from the province of Central Java, Indonesia. After cleaning, the green mussel and crab shells were oven-dried at 100 °C for 2 h. The dried green mussel and crab shells were reduced using a crusher machine and sieved with 100 mesh screens. This process produced the green mussel and crab shell powders. The calcination process was carried out on the green mussel and crab shell powders using a Thermolyne Furnace Chamber F6010 at a temperature of 900 °C for 5 h. The green mussel and crab shell powders before and after calcination were characterized using SEM-EDX and XRD methods.

In this study, Precipitated Calcium Carbonate (PCC) was synthesized using calcined green mussel and crab shell powders. Seventeen grams of calcined crab shell powder were mixed with 300 mL of 2M HNO_3_.

To produce a homogeneous mixture, stirring was carried out using a magnetic stirrer at 60 °C for 30 min with a rotation speed of 30 rpm. After 30 min, NH_4_OH was added until the pH of the solution reached 12, which was then filtered using Whatman 42 filter paper. The filtrate was precipitated by slowly flowing CO_2_ gas. The resulting milky white precipitate was then washed and filtered with distilled water to a pH of 7 and then dried at 110 °C for 2 h. The exact process was also carried out to produce PCC made from calcined green mussel shell powder [19].

SEM-EDX (SEOL JSM–6510LA) method was used to determine PCC’s morphology and chemical elements made of green mussel and crab shells. The Shimadzu XRD-7000 was also used to analyze PCC’s resulting phase and crystallite size from the green mussel and crab shells. Crystalline phases presented in the samples were identified with the help of the Joint Committee on Powder Diffraction Standards (JCPDS). The Rietveld analysis was conducted using the High Score Plus software version 3.0e from PANalytical X’Pert, Cambridge, UK. The description of the diffraction line profiles at Rietveld refinement was achieved using the pseudo-Voigt function. For comparison, SEM-EDX dan XRD tests were held to commercial-grade calcium carbonate gained from Merck.

## 3. Results and Discussion

### 3.1. Characterization of Green Mussel and Crab Shell Powders

**Figure 1:**
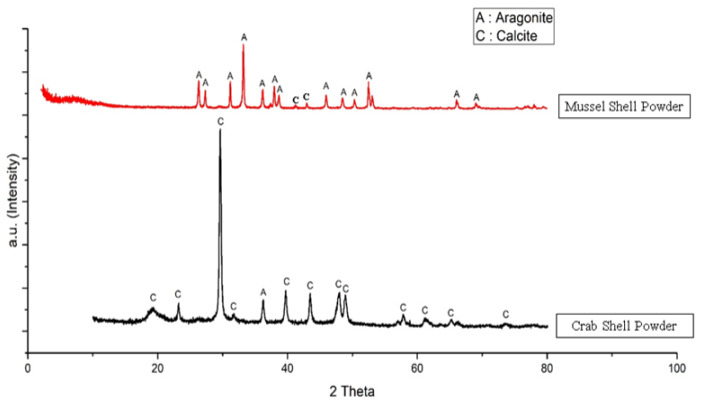
XRD diffraction pattern of green mussel and crab shell powders.

Figure 1 shows a graphic comparison of the XRD test results on green mussel and crab shell powder. Calcite and aragonite crystalline phases were found in powdered green mussel and crab shells. Following JCPDS card number 05-0586, the phase of calcite crystals in mussel and crab shell powder is generally denoted as 2θ: 29.404, 39.399, and 43.143. Meanwhile, the aragonite crystal phase is shown at 2θ: 26.312, 31.176, and 33.180 (JCPDS Card No. 05-0453). In this study, the powder of green mussel shells was dominated by an aragonite crystal phase with a little calcite crystal phase. This contrasts with the crystalline phase found in powdered crab shells dominated by the calcite crystalline phase with a little aragonite crystalline phase.

**Figure 2:**
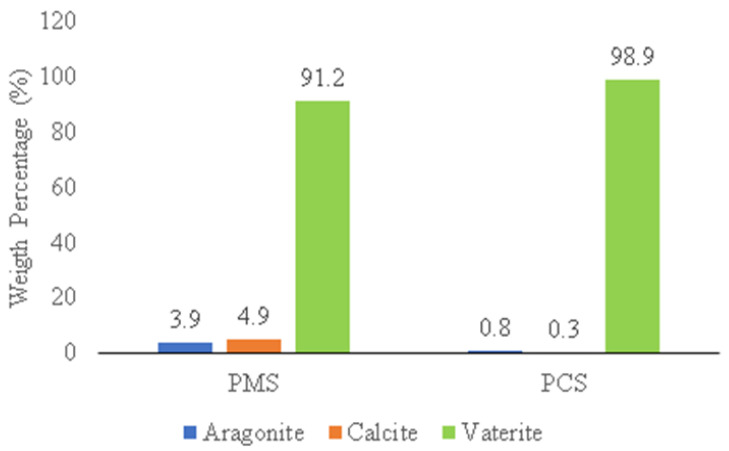
Comparison of weight percentage (%) of crystal phase on green mussel shell and crab shell powder.

**Figure 3:**
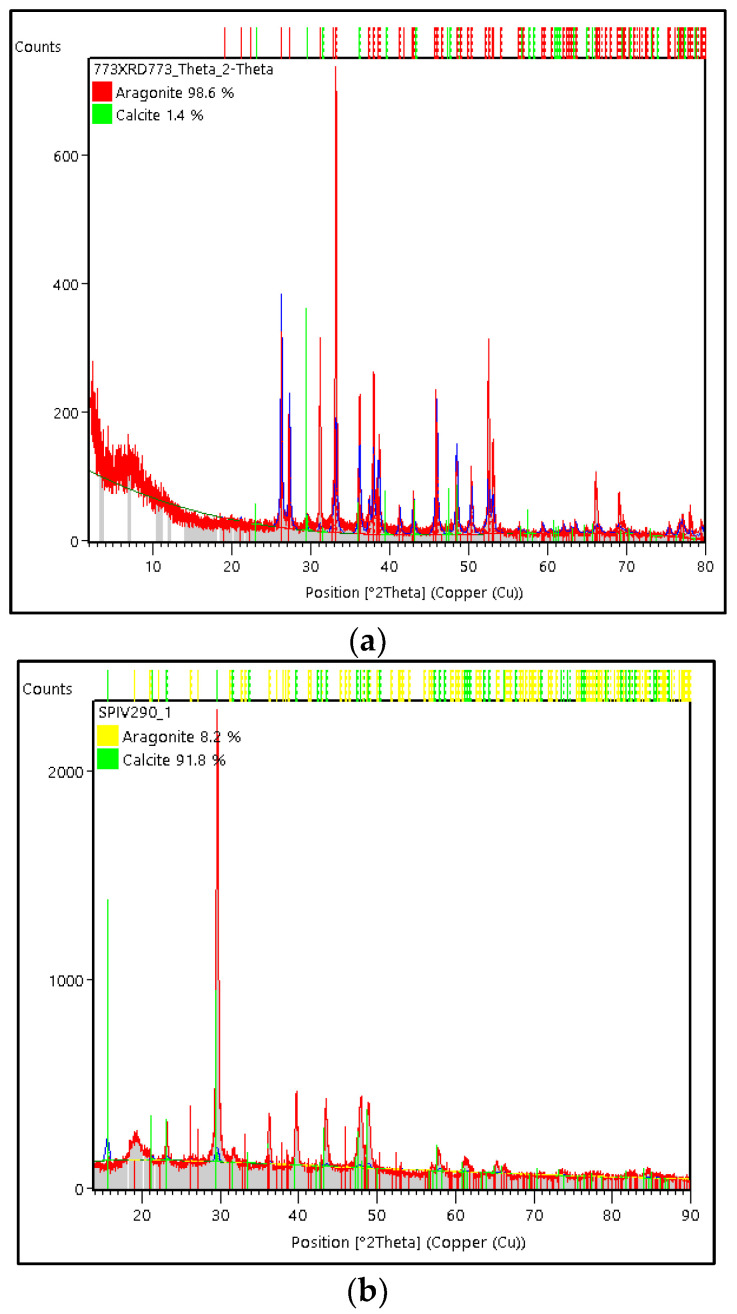
XRD diffraction patterns of (**a**) green mussel shells powder and (**b**) crab shell powder.

The comparison of the weight percentage (%) phase of calcite and aragonite crystals on green mussel and crab shell powders is shown in Figure 2. Weight percentage (%) of calcite and aragonite crystals were generated from a Rietveld analysis carried out using High Score Plus software version 3.0e, as shown in Figure 3a,b. In the green mussel shells powder, the aragonite crystal had a weight percentage (%) and crystallite size of 98.6% and 59 nm, respectively, with an orthorhombic crystal system. Meanwhile, the calcite phase in the crab shells powder showed a trigonal crystal system with a weight percentage (%) and crystallite size of 91.8% and 19 nm.

**Figure 4:**
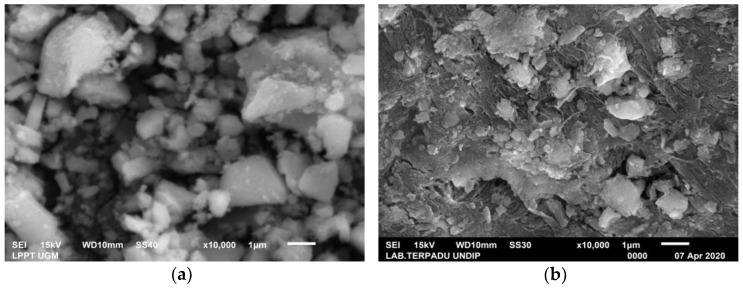
SEM images of (**a**) green mussel shells powder and (**b**) crab shell powder.

The XRD test results on green mussel and crab shell powders showed the crystal phase of aragonite and calcite dominance, respectively. This is supported by the SEM test results shown in Figure 4a,b. Scanning Electron Microscopy (SEM) was used to study the morphology of the synthesis product [20,21,22]. The morphology of the green mussel shell powder is irregular in shape and resembles a small branching rod, which represents the characteristics of aragonite crystals [23]. The common morphologies of aragonite are rod-like, multilayered, pseudohexagonal, needle-like, and dendrite-like [24].

Meanwhile, the morphology of the crab shell powder shows a predominance of a nail head and cubic-like structure, which is the characteristic of calcite crystals [25,26].

Energy Dispersive X-ray Analysis (EDX), referred to as EDS or EDAX, is used to determine the mineral content of green mussel and crab shell powder. Table 1 shows a summary of the EDX results of green mussel and crab shell powder, with the major components found to be Ca, C, and O. The EDX test results show the presence of Na in green mussel shells, although in small amounts. Meanwhile, in crab shells, Na, Mg, P, and Zr are found in addition to Ca, C, and O elements.

### 3.2. Characterization of Green Mussel and Crab Shell Powders after Calcination

**Figure 5:**
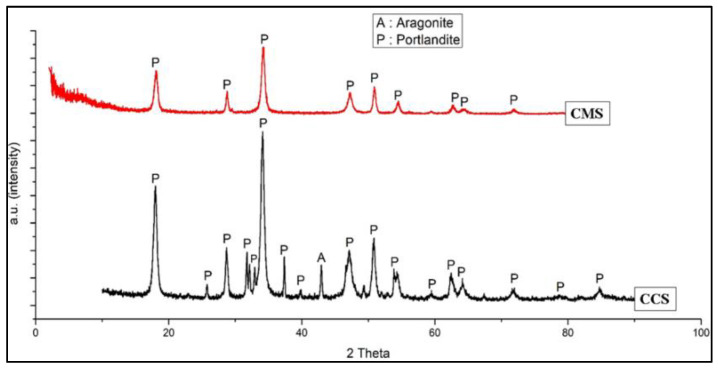
XRD diffraction pattern of CMS and CCS.

In this study, the calcination process was carried out on the powder of the green mussel and crab shells using a Thermolyne Furnace Chamber F6010 at a temperature of 900 °C for 5 h. Here, calcination produces Calcined Green Mussel Shells (CMS) and Calcined Crab Shells (CCS). Figure 5 shows a graphic comparison of the results of XRD testing on CMS and CCS with the dominance of the portlandite crystal phase. Portlandite was observed at 2θ: 18.0123, 34.1015, and 50.7966 (JCPDS Card No. 44-1481). Calcination is generally used to remove organic compounds and impurities in green mussel powder and crab shells. In addition, calcination in green mussel powder and crab shells with a temperature of 900 °C for 5 h to convert the CaCO_3_ compound into CaO is given in the reaction equation [27]:

CaCO_3_ (S) → CaO (S) + CO_2_ (g) (1)

After 5 h, cooling down to room temperature was held by turning off the furnace without opening it (cooling in the furnace). It was done to avoid the possibility of damage to the furnace walls due to the sudden change of temperature. After reaching room temperature, the furnace was opened to collect the green mussel and crab shell powders.

During the cooling process, there was contact between the CaO compound in the green mussel and crab shell powders with air containing water vapor so that hydration could occur, and portlandite or Ca(OH)_2_ was formed through the reaction equation [27]:

2CaO (s) + 2H_2_O (g) → 2Ca(OH)_2_ (s) (2)

This is what causes the calcite and aragonite crystals in the powder of green mussel and crab shells to transform into calcium hydroxide or portlandite (Ca(OH)_2_) after calcination [27,28].

**Figure 6:**
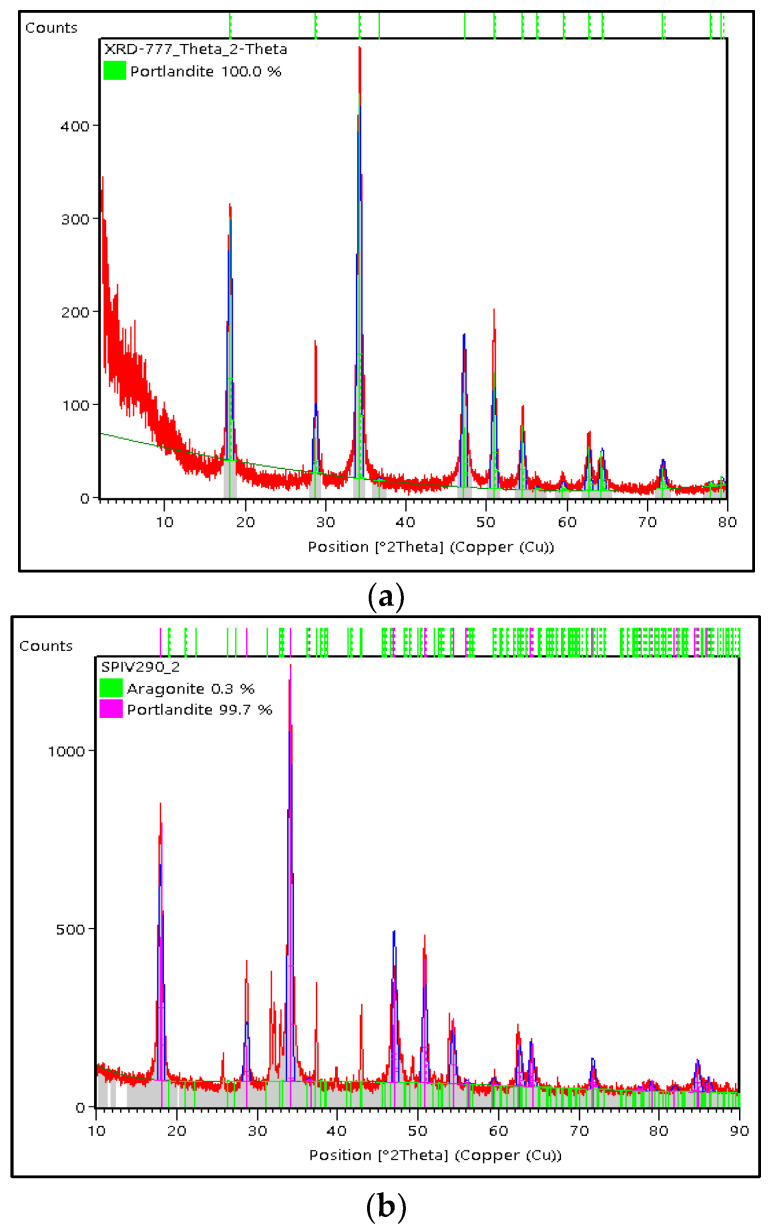
X-ray diffraction patterns of (**a**) CMS and (**b**) CCS.

**Figure 7:**
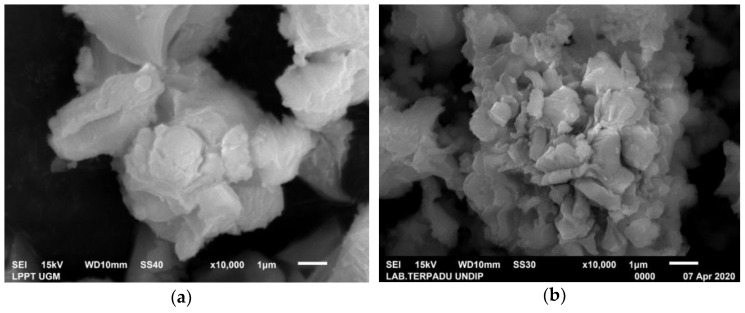
SEM images of (**a**) CMS and (**b**) CCS.

In this study, only a portlandite crystal phase was found in CMS, while in CCS, there was still a small amount of aragonite crystal phase. The weight percentage (%) of the portlandite crystal phase was generated from a Rietveld analysis using High Score Plus software version 3.0e, as shown in Figure 6a,b. In CMS, the portlandite crystal has a weight percentage (%) and crystallite size of 100% and 14 nm, respectively, with the trigonal crystal system. Meanwhile, the portlandite phase on CCS shows a trigonal crystal system with a weight percentage (%) and crystallite size of 99.7% and 12 nm, respectively. Apart from the portlandite phase, aragonite crystals in CSS were also found with a weight percentage (%) of 0.3%. The XRD test results on the CMS and CCS are supported by the SEM test results, as shown in Figure 7a,b. As a result of the study, portlandite crystallized in the shape of imperfect cubic and irregular shapes. The portlandite crystals’ morphology in this study is consistent with the literature [29,30].

Table 2 shows a summary of the EDX results on CMS and CCS with the major components found being Ca, C, and O. The EDX test results show that the Na element in green mussel shells powder before calcination was not found in CMS. Meanwhile, in CCS, there were still elements of Na, Mg, and P, and Ca, C, and O. After calcination, the Ca content in CMS and CCS was more than the Ca content in the green mussel and crab shell powders. Meanwhile, the C content in CMS and CCS was less than the C content in the green mussel and crab shell powders. This happened because of the decomposition process, where C bound to O to form CO_2_ gas and Ca bound to O to form CaO.

### 3.3. Characterization of Green Mussel and Crab Shell Powders after Precipitation Process

In this study, the Precipitated Calcium Carbonate (PCC) produced from green mussel shells powder was labeled as PMS, whilst the Precipitated Calcium Carbonate (PCC) produced from crab shells powder was labeled as PCS.

**Figure 8:**
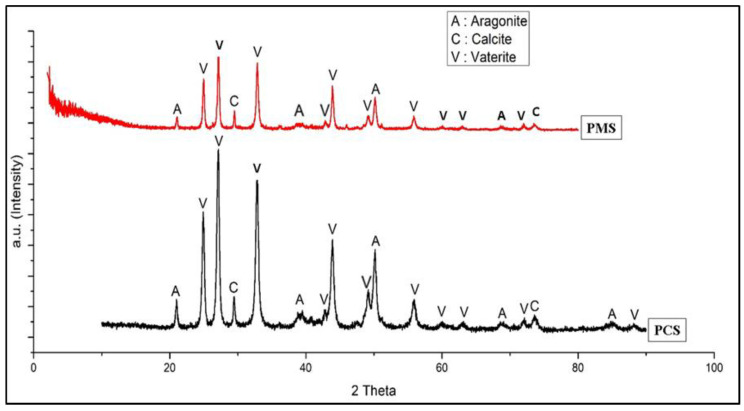
XRD diffraction pattern of PMS and PCS.

Figure 8 shows a comparison graph of the XRD test results on PMS and PCS. The crystalline phases of vaterite, calcite, and aragonite were found in PMS and PCS. Following JCPDS card number 13-0192, the phase of the vaterite crystal in PMS and PCS is denoted by 2θ: 24.9011, 27.0705, and 32.7760. In addition to that, there were calcite and aragonite crystal phases that correspond to the JCPDS card numbers 05-0586 and 05-0453. In this study, PMS and PCS were dominated by the vaterite crystalline phase, with a few calcite and aragonite crystalline phases.

In this study, CMS and CCS with portlandite or Ca(OH)_2_ as the dominant phase was converted to PMS and PCS, which had CaCO_3_ (vaterite) as the predominant crystal phase precipitation process using CO_2_ gas. CaCO_3_ may be obtained when portlandite (Ca(OH)_2_) was exposed to atmospheric carbon dioxide (CO_2_). The transformation of portlandite to CaCO_3_ is under the equation [30]:

Ca(OH)_2_ (s) + CO_2_ (g) → CaCO_3_ (s) + H_2_O (g) (3)

The formation of the vaterite dominant phase in PMS and PCS can occur due to several things, such as the reaction temperature, Ph, and CO_2_ flow rate at the time of carbonation [31,32]. In this study, the carbonation process was carried out at a temperature of 30 °C, pH 12 and a low flow rate of CO_2_. The dominant vaterite phase was caused by an increase in the concentration of CO_2_ gas. That happens because the amount of CO_2_ gas added will increase the solubility of CO_2_ gas in the solution. The increasing solubility of CO_2_ gas in the solution causes an increase in the supersaturation of the solution so that vaterite is formed as the dominant crystalline phase [33].

**Figure 9:**
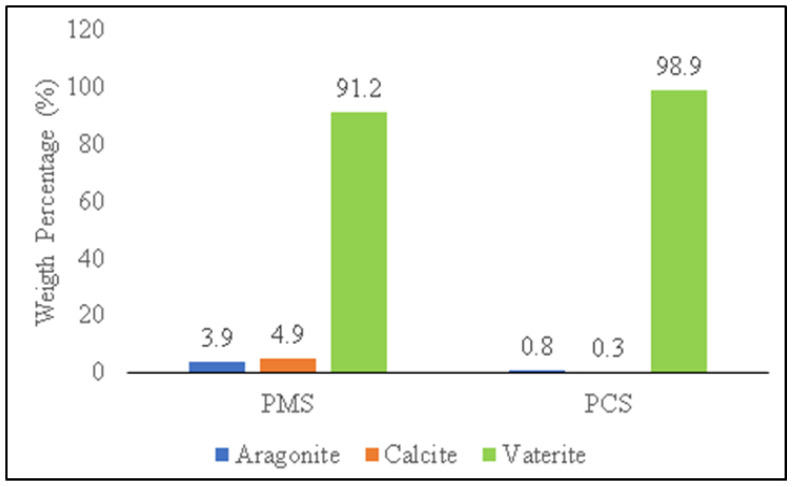
Weight percentage (%) comparison of crystal phase on PMS and PCS.

A comparison of the weight percentage (%) phase of vaterite, calcite, and aragonite crystals on PMS and PCS is shown in Figure 9.

**Figure 10:**
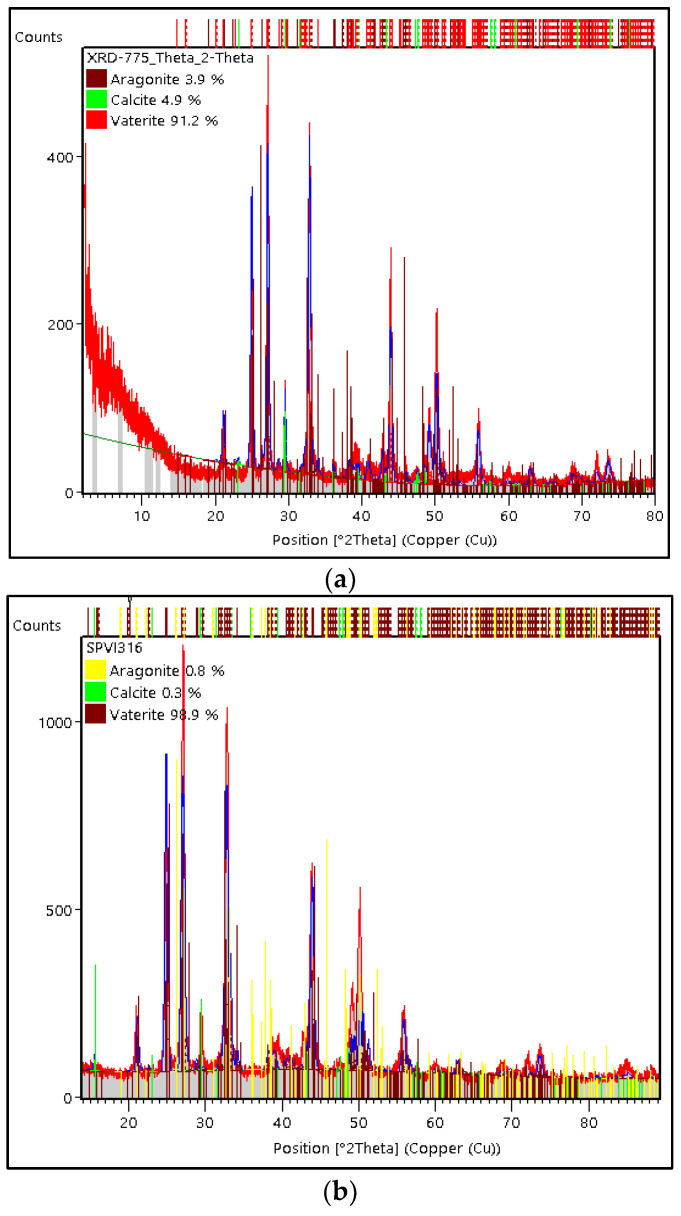
X-ray diffraction patterns of (**a**) PMS and (**b**) PCS.

The weight percentage (%) was generated from Rietveld analysis conducted using High Score Plus software version 3.0e, as shown in Figure 10a,b. In PMS, the vaterite crystal had a weight percentage (%) and crystallite size of 91.2% and 34 nm, respectively, with the monoclinic crystal system. Meanwhile, the weight percentage (%) on calcite and aragonite was 4.9% and 3.9%, respectively. The vaterite phase of PCS shows the monoclinic crystal system with a weight percentage (%) and crystallite size of 98.9% and 21 nm, respectively. Apart from the vaterite phase, PCS also found aragonite and calcite crystals with a weight percentage (%) of 0.8% and 0.3%, respectively.

**Figure 11:**
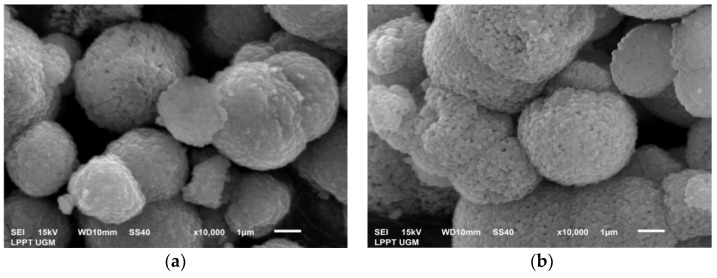
SEM images of (**a**) PMS and (**b**) PCS.

The test results on PMS and PCS showed the dominance of the vaterite crystal phase. The results of the SEM tests support these results carried out as shown in Figure 11a,b. As a result of the study, vaterite was crystallized in a spherical shape. The vaterite crystals’ morphology in this study is consistent with the literature [34,35]. Table 3 summarizes the EDX results on PMS and PCS, and the major components found are Ca, C, and O. After precipitation, PMS and PCS experience a decrease in Ca content due to the addition of CO_2_ gas into the solution. This is proved by the increase in the C and O content. EDX testing showed that only Ca, C, and O were found in PMS. Meanwhile, PCS found N elements other than Ca, C, and O. Element N found in PCS was caused by mixing less homogeneous Ca(OH)_2_ with 2M HNO_3_, leading to a less perfect reaction of NO_3_^−^ ions.

### 3.4. The Comparison of the Characterization of PMS, PCS, and Commercial CaCO_3_

**Figure 12:**
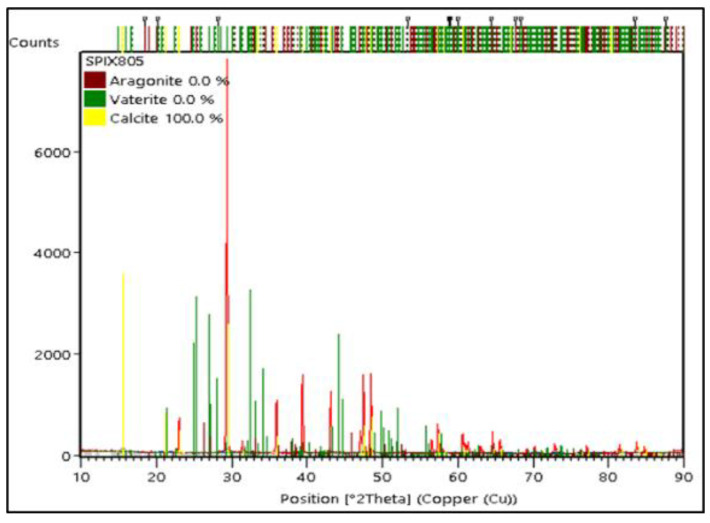
X-ray Diffraction Patterns of Commercial CaCO_3_.

**Figure 13:**
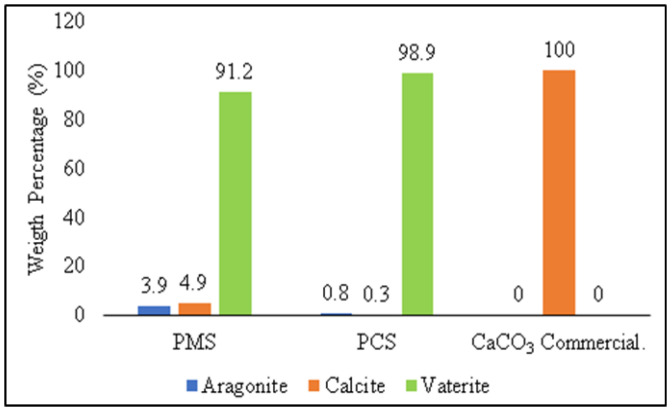
Weight Percentage Comparison (%) of Crystal Phase on PMS, PCS, and Commercial CaCO_3_.

The XRD test results’ analysis on commercial CaCO_3_ were carried out with Rietveld analysis using High Score Plus software version 3.0e to determine the crystal phase formed, weight percentage (%), and crystallite size, as shown in Figure 12. In commercial CaCO_3_, there was only a calcite phase as a single phase with a trigonal crystal system. The weight percentage (%) and crystallite size of calcite on commercial CaCO_3_ were 100% and 15 nm, respectively. The crystallite size for commercial CaCO_3_ was 15 nm, while the crystallite size for PMS and PCS were 34 nm and 21 nm, respectively. A comparison of the weight percentage (%) for each crystal phase found in PMS, PCS, and commercial CaCO_3_ is shown in Figure 13. In commercial CaCO_3_, there is only calcite as a single phase. Whereas in PMS and PCS, vaterite is the dominant crystalline phase.

In this study, after the carbonation process of the calcined powder was produced from green mussel and crab shells, vaterite was the dominant crystalline phase in the calcium carbonate polymorph. Similar findings were reported by Prihanto et al. (2022). A quantitative XRD Rietveld examination of PCC products generated from green mussel shells revealed the presence of vaterite (55.20 wt %) and calcite (44.40 wt %) minerals following the carbonation of the calcined powder product [36]. According to Ismail et al. (2021), no polymorphic difference was noticed between the PCC product and the CO_2_ stream in contact with the reaction solution. Importantly, PCC-800 and PCC-900 products include a high concentration of vaterite, a promising biomaterial for use in drug delivery systems [19].

**Figure 14:**
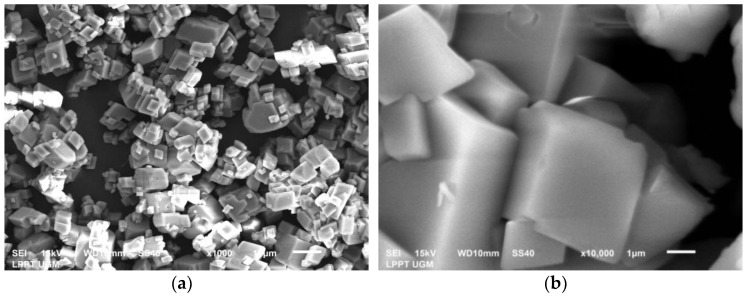
SEM images with different magnifications of (**a**) 1000 and (**b**) 10,000 times of commercial CaCO_3_.

The crystalline phase of calcium carbonate is generally in the form of calcite, aragonite, and vaterite. The different morphological forms are due to different synthesis conditions [37,38]. Calcite has stable structures, mechanical properties, and thermodynamic properties, so it is widely used in biomedical applications [39]. Aragonite is formed in the orthorhombic system and is biocompatible. Aragonite can be broken down, combined, and it can also replace bone. Aragonite is denser than calcite and has also been used for biomedical applications [40,41]. Vaterite has low stability and belongs to the hexagonal crystal system. In contact with water, vaterite can slowly dissolve and recrystallize to a stable form [42]. Due to its nontoxicity, good biocompatibility and affinity, low cost, and ease of large-scale production, vaterite can be used as an ideal nominee for biomedical applications [35,42].The XRD results on commercial CaCO_3_ are supported by the SEM test results shown in Figure 14. Commercial CaCO_3_ has a morphology with a cube-like shape, which is characteristic of calcite [37]. Meanwhile, PMS and PCS have spherical morphology, which is characteristic of vaterite crystals. The results of this study are the same as the research conducted by Hamester et al. (2012) [43].

**Figure 15:**
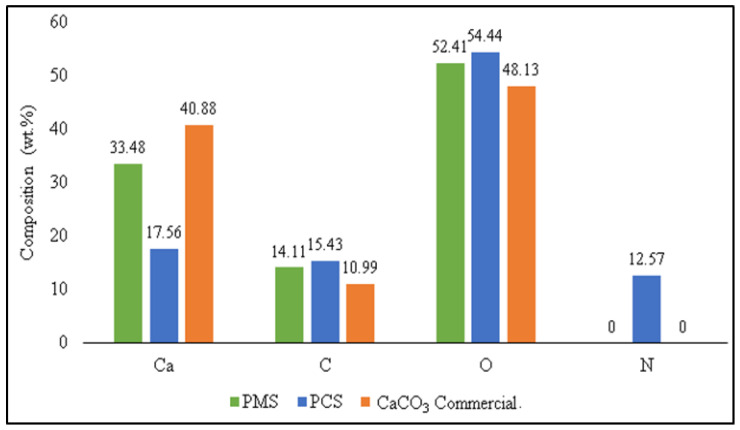
Chemical Composition Comparison of PMS, PCS, and Commercial CaCO_3_.

The EDX test results show that PMS and commercial CaCO_3_ only contain Ca, C, and O. Meanwhile, PCS found the presence of N elements besides Ca, C, and O. The results of the research conducted by [44] stated that the chemical composition of commercial CaCO_3_ (calcite) consists of only Ca, C, and O. The common elements such as Ca, C, and O were present in the CaCO_3_ derived from green mussel shells (PMS), indicating the success of the precipitation method [45]. The comparison of the chemical composition of PMS, PCS, and commercial CaCO_3_ is shown in Figure 15. In this study, the Ca content in commercial CaCO_3_ was higher than the Ca content in PMS and PCS. While the C and O content in commercial CaCO_3_ was lower than the C and O content in PMS and PCS. In this study, the chemical composition of PMS was better than the chemical composition of PCS. Moreover, the chemical composition of PMS was almost the same as the chemical composition of commercial CaCO_3_. In addition, in PMS, there were no impurities.

A similar result was stated by Ismail et al. (2021). The EDX analysis revealed that PCC samples derived from green mussel shells (PCC-800 and PCC-900) included primarily Ca, C, and O. Introducing a stream of CO_2_ into the solution caused a decrease in the Ca concentration of PCC samples. Conversely, the carbonization process raises the C and O concentration [19]. Prihanto et al. (2022) discovered differences showing that in addition to Ca, C, and O, PCC derived from green mussel shells contained Zn and Cu. Based on the EDX analysis, secondary reactions involving the formation of solid solutions correlating to the existence of Cu and Zn may also exist, especially along the adsorption–precipitation boundary [36].

## 4. Conclusions

The use of green mussel and crab shells after industrial and consumption activities has many benefits. It has the potential to be applied in various fields, including in the field of biomaterials. This research shows that the calcination and precipitation processes significantly affect the chemical composition, crystal phase, crystal size, and crystal system of the CaCO_3_ obtained. The crystalline phases of aragonite and calcite in green mussel and crab shell powders were converted to Ca(OH)_2_ or portlandite by calcination. In addition, calcination can remove organic compounds and reduce the impurity content in green mussel powder and crab shells. The chemical composition of PMS and commercial CaCO_3_ shows that they existed only as Ca, C, and O. In contrast, in PCS, it was found that the element N is an impurity in addition to Ca, C, and O. The results of this study show that PMS can be used as a candidate for biomaterials because the chemical composition in PMS is almost the same as the chemical composition in commercial CaCO_3_, where no impurities were found. In addition, the dominance of the vaterite crystal phase in PMS is a distinct advantage for biomaterial applications because it has nontoxicity, good biocompatibility and affinity, a low cost, and ease of large-scale production.

The crystallite size produced in PMS is more prominent than commercial CaCO_3_. The crystallite size of vaterite in PMS is 34 nm, while the crystallite size of calcite in commercial CaCO_3_ is 15 nm.

## Figures and Tables

**Table 1:** The summary of EDX results on the green mussel and crab shells.

Element	Composition (wt.%)	
	Crab Shells	Green Mussel Shells
C	29.44	17.56
O	38.97	53.09
Ca	24.49	28.85
Na	0.38	0.50
Mg	1.4	-
P	3.33	-
Zr	1.99	-
Total	100	100

**Table 2:** The summary of EDX results on CMS and CCS.

Element	Composition (wt.%)			
	Green Mussel Shells		Crab Shells	
	Powders	CMS	Powders	CCS
C	17.56	7.13	29.44	7.38
O	53.09	51.21	38.97	47.99
Ca	28.85	41.66	24.49	37.18
Na	0.5	-	0.38	0.58
Mg	-	-	1.4	3.89
P	-	-	3.33	2.98
Zr	-	-	1.99	-
Total	100	100	100	100

**Table 3:** The summary of EDX results on PMS and PCS.

Element	Composition (wt.%)					
	Green Mussel Shells			Crab Shells		
	Powders	CMS	PMS	Powders	CCS	PCS
C	17.56	7.13	14.11	29.44	7.38	15.43
O	53.09	51.21	52.41	38.97	47.99	54.44
Ca	28.85	41.66	33.48	24.49	37.18	17.56
Na	0.5	-	-	0.38	0.58	-
Mg	-	-	-	1.4	3.89	-
P	-	-	-	3.33	2.98	-
Zr	-	-	-	1.99	-	-
Total	100	100	100	100	100	100

## Data Availability

Data are contained within the article.
